# Association of Platelet to Lymphocyte and Neutrophil to Lymphocyte Ratios with In-Hospital Mortality in Patients with Type A Acute Aortic Dissection

**DOI:** 10.21470/1678-9741-2018-0343

**Published:** 2019

**Authors:** Cihan Bedel, Fatih Selvi

**Affiliations:** 1 University of Health Sciences, Antalya Training and Research Hospital, Department of Emergency Medicine, Antalya, Turkey.

**Keywords:** Aneurysm, Dissecting, Leukocyte Count, Lymphocytes, Hospital Mortality, Blood Platelets, Risk Factors, Hospitalization

## Abstract

**Objective:**

To evaluate the relationship between neutrophil to lymphocyte ratio (NLR) and platelet to lymphocyte ratio (PLR) with in-hospital mortality in type A acute aortic dissection (AAD).

**Methods:**

A total of 96 patients who presented to the emergency department between January 2013 and June 2018 with a diagnosis of type A AAD were enrolled in this study. White blood cell count subtypes such as NLR and PLR were calculated at the time of admission. The end point was in-hospital mortality.

**Results:**

Of the 96 type A AAD patients included in this analysis, 17 patients (17.7%) died during hospitalization. NLR and PLR were significantly elevated in patients with type A AAD (*P*<0.001 and <0.001, respectively). Based on the receiver operating characteristic curve, the best NLR cut-off value to predict in-hospital mortality was 9.74, with 70.6% sensitivity and 76.8% specificity, whereas the best PLR cut-off value was 195.8, with 76.5% sensitivity and 78.1% specificity.

**Conclusion:**

Admission NLR and PLR levels were important risk factors and independently associated with in-hospital mortality of type A AAD patients.

**Table:** 

Abbreviations, acronyms & symbols	
AAD	= Acute aortic dissection
AUC	= Area under the curve
CI	= Confidence interval
CRP	= C-reactive protein
CT	= Computed tomography
F	= Female
M	= Male
MPV	= Mean platelet volume
NLR	= Neutrophil to lymphocyte ratio
PCT	= Plateletcrit
PDW	= Platelet distribution width
PLR	= Platelet to lymphocyte ratio
RDW	= Red cell distribution width
ROC	= Receiver operating characteristic
SD	= Standard deviation
SPSS	= Statistical Package for the Social Sciences
WBC	= White blood cell

## INTRODUCTION

Type A acute aortic dissection (AAD) is a destructive cardiovascular condition with a mortality rate of 1-2% per hour, after the onset of symptoms^[1]^. Determination of risk factors for prognosis is of great value for risk classification in patients with type A AAD. In recent years, chest pain, hypotension, and inflammatory biomarkers, such as C-reactive protein (CRP), have been shown to be related with the prognosis in patients with AAD^[2,3]^.

White blood cell (WBC) count and its subtypes are widely known as systemic inflammation markers that have been associated with bad clinical outcomes in various cardiovascular diseases^[4,5]^. Moreover, there are few data regarding the association of neutrophil to lymphocyte ratio (NLR) and platelet to lymphocyte ratio (PLR) with in-hospital mortality in patients with type A AAD. Therefore, we conducted a retrospective clinical study to evaluate the relationship between admission NLR and PLR with in-hospital mortality in patients with type A AAD.

## METHODS

This retrospective study was approved by the local ethical committee and the tenets of the Declaration of Helsinki were followed. After the approval, we carried out a retrospective hospital record review of the data of patients with suspected Stanford type A AAD in our emergency department between January 2013 and June 2018 and these were enrolled in this study. The diagnosis of AAD was confirmed by aorta angiography with multidetector computed tomography (CT) scanning. Inclusion criteria of this study were: (1) type A AAD within 24 hours after symptom onset and (2) age over 18 years. Patients with missing clinical, laboratory, or radiographic data and those who had chronic liver and renal disease, hematological diseases, inflammatory or autoimmune diseases, malignant tumor, and prior history of aortic dissection were excluded. Clinical baseline characteristics, results of CT scan, hematologic laboratory data, and all clinical outcomes were obtained from a review of the each patient’s chart in the database of our hospital. The reason and strategy of surgical techniques were determined by the surgeons in the department of cardiovascular surgery in our hospital.

The laboratory results were evaluated using the first venous blood samples taken on admission to the emergency department. Samples were analyzed within one hour of collection using an automated blood cell counter (Coulter® LH 780 Hematologic Analyzer, Beckman Coulter Inc. Brea, USA). Reference values were 2.1-6.1×10^3^/mm^3^ for neutrophils, 1.3-3.5×10^3^/mm^3^ for lymphocytes, 156- 373×10^3^/mm^3^ for platelets. The NLR was calculated as the ratio of neutrophil count to lymphocyte count, and the PLR was calculated as the ratio of platelet count to lymphocyte count. The study end point was identified as all-cause mortality during hospitalization.

### Statistical Analysis

We performed all statistical analyses with the Statistical Package for the Social Sciences (SPSS) software (SPSS, Inc., Chicago, Illinois, USA), version 18.0. Results are presented as mean±standard deviation (SD) with interquartile range unless otherwise stated. Variations between interventions are reported as mean differences with 95% confidence intervals (95% CI). Baseline characteristics were compared between survivor and non-survivor patients using unpaired Student’s t-tests, Wilcoxon-Mann-Whitney tests for continuous data, and Chi-square tests for categorical data. Categorical variables were defined as a percentage. Receiver operating characteristic (ROC) analysis was performed to determine the cut-off value for NLR and PLR in predicting in-hospital mortality with high sensitivity and specificity. To define the independent predictors associated with in-hospital mortality in AAD patients, univariate analysis and multiple logistic regression analysis were used to identify the factors related to in-hospital mortality.

## RESULTS

**Fig. 1:**
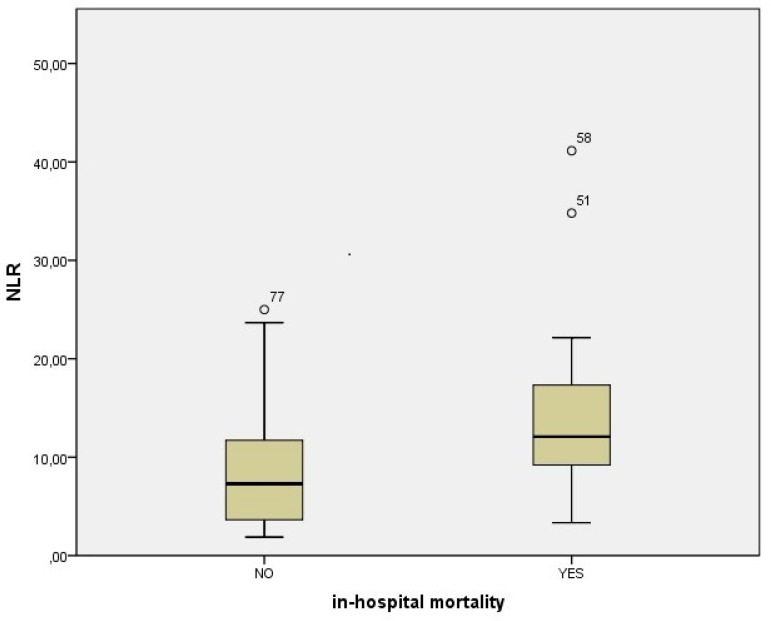
Comparison of neutrophil to lymphocyte ratio (NLR) between survivor and non-survivor patients admitted with type A acute aortic dissection.

**Fig. 2:**
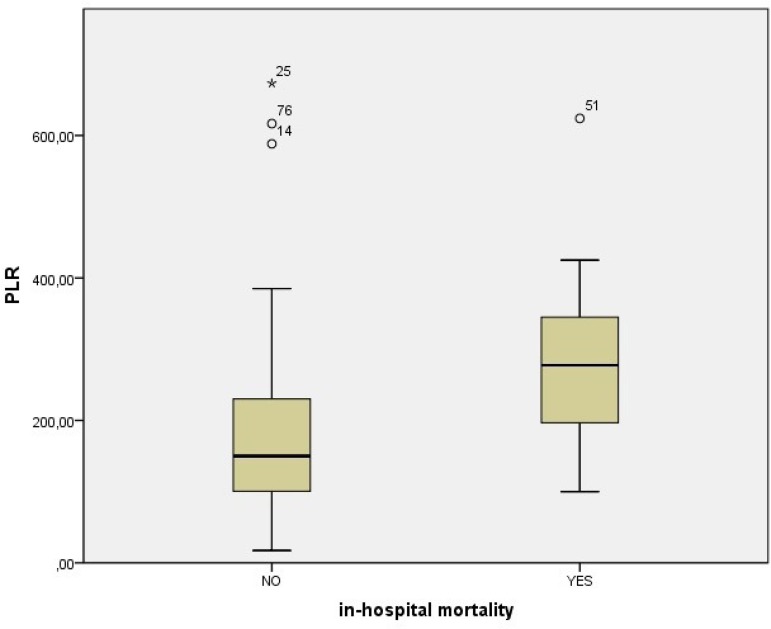
Comparison of platelet to lymphocyte ratio (PLR) between survivor and non-survivor patients admitted with type A acute aortic dissection.

A total of 96 patients with a diagnosis of type A AAD were included in the present study. There were 78 (81.2%) male and 18 (18.8%) female patients, with a male to female ratio of 4.3. The mean patients’ age was 63.7±13.4 years. In-hospital mortality rate was found to be 17.7% (17 of 96 patients). Demographic and laboratory characteristics of survivors and non-survivors were summarized in Table 1. In all the non-survivors, the levels of WBC, neutrophil, PLR, and NLR were significantly increased and the levels of lymphocyte were significantly decreased (*P*<0.05), and they were slightly older than survivors. The NLR was 8.5±5.7 in survivor patients and 15.5±10.1 in non-survivor patients (*P*<0.001, Figure 1). The PLR was 182.3±122.9 in survivor patients and 279.3±131.6 in non-survivor patients (*P*<0.001, Figure 2). The NLR values of the patients with and without surgery were 9.1±6.4 and 14.9±11.1, respectively, and the difference between them was not statistically significant (*P*=0.058); the PLR values of the patients with and without surgery were 195.8±133.2 and 231.2±87.1, respectively, and the difference between them was not statistically significant (*P*=0.109). Furthermore, there were no significant differences between the groups in terms of hemoglobin, platelet count, platelet distribution width (PDW), mean platelet volume (MPV), plateletcrit (PCT), red cell distribution width (RDW), and RDW-to-platelets ratio levels.

**Table 1:** Clinical characteristics of study population.

Parameter	All patients (n=96)	Survivors (n=79)	In-hospital death (n=17)	*P*-value
Age (years; mean±SD)	63.7±13.6	62.3±12.8	70.3±14.6	0.030
Gender (M/F;%)	78/18(81.2/11.8)	66/13(83.5/16.5)	12/5(70.6/29.4)	0.210
WBC count (10^3^/mm^3^; mean±SD)	12.3±4.7	11.7±4.1	15.3±6.1	0.016
Neutrophil count (10^3^/mm^3^; mean±SD)	9.9±4.5	9.2±3.9	10.1±5.8	0.006
Lymphocyte count (×10^3^/mm^3^; mean±SD)	1.3±0.6	1.4±0.7	0.9±0.4	0.019
Platelet count (×10^3^/mm^3^; mean±SD)	220.2±106.9	213.2±107.9	252.5±99.1	0.117
PDW (fL; mean±SD)	16.1±2.1	16.1±1.9	15.7±2.4	0.836
MPV (fL; mean±SD)	9.2±1.3	9.2±1.4	9.2±1.3	0.744
PCT (%; mean±SD)	0.2±0.1	0.2±0.1	0.2±0.1	0.849
RDW (%; mean±SD)	15.3±2.1	15.4±2.1	15.1±1.6	0.584
RDW to platelets ratio	0.1±0.07	0.1±0.08	0.07±0.03	0.081
PLR	199.5±129.2	182.3±122.9	279.3±131.6	<0.001
NLR	9.8±7.2	8.5±5.7	15.5±10.1	<0.001
Surgery (n;%)	86(89.6)	79(100)	7(41.2)	<0.001

F=female; M=male; MPV=mean platelet volume; NLR=neutrophil to lymphocyte ratio; PCT=plateletcrit; PDW=platelet distribution width; PLR=platelet to lymphocyte ratio; RDW=red cell distribution width; SD=standard deviation; WBC=white blood cell

To define the factors which can predict type A AAD related in-hospital mortality, multiple regression analysis was applied using the significant variables from the univariate analysis (*P*<0.05) (Table 2). It was found that increased WBC, neutrophil, PLR, NLR, slightly older age, surgical intervention, and decreased lymphocyte were independent predictors of in-hospital mortality in type A AAD (*P*<0.05) (Table 2).

**Table 2:** Multiple regression analysis of risk factors that affected type A AAD related in-hospital mortality.

Variables	Odds ratio	95% CI	*P*
Age (years)	1.071	1,016 to 1.130	0.03
WBC count (×10^3^/mm^3^)	2.221	0.823 to 5.997	0.009
Neutrophil count (×10^3^/mm^3^)	0.620	0.203 to 1.895	0.005
Lymphocyte count (×10^3^/mm^3^)	0.063	0.003 to 1,169	0.027
PLR	1.004	0.998 to 1.010	0.010
NLR	0.873	0.664 to 1.148	0.002
Surgery	2.429	1.376 to 4.286	<0.001

AAD=acute aortic dissection; CI=confidence interval; NLR=neutrophil to lymphocyte ratio; PLR=platelet to lymphocyte ratio; WBC=white blood cell

**Fig. 3:**
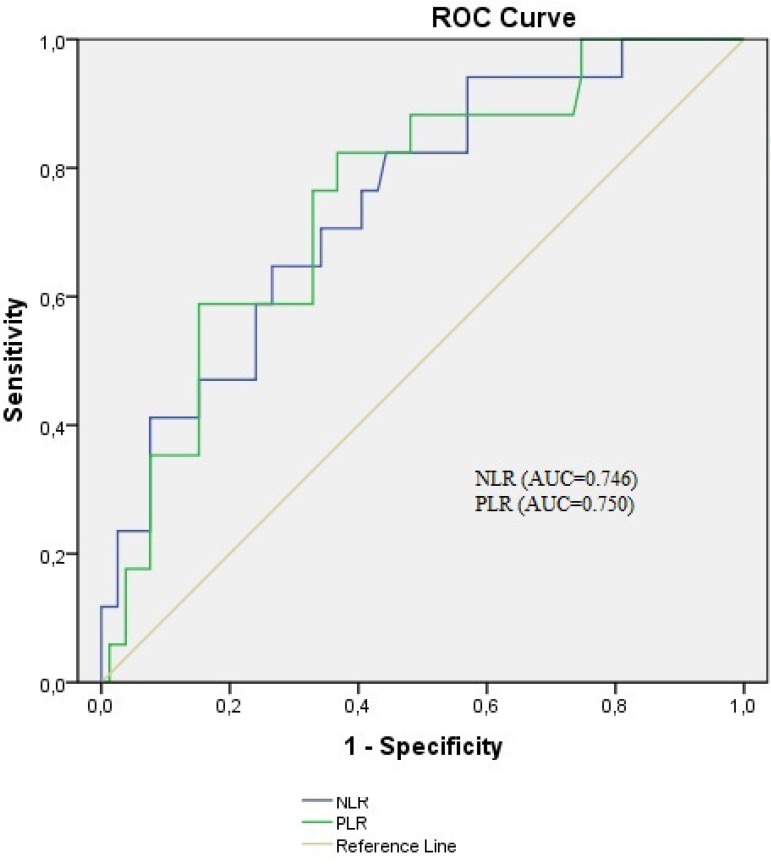
Receiver operating characteristic (ROC) curve of NLR and PLR for predicting in-hospital mortality in patients with type A acute aortic dissection. AUC=area under the curve; NLR=neutrophil to lymphocyte ratio; PLR=platelet to lymphocyte ratio

ROC curve analysis was performed to detect the best cut-off value of NLR and PLR in the prediction of in-hospital mortality in patients with type A AAD (Figure 3). An NLR >9.74 was given an area under the curve (AUC) value of 0.746 (95% CI 0.623-0.870, <0.001). Furthermore, an NLR >9.74 demonstrated a sensitivity of 70.6% and specificity of 76.8% for the prediction of in-hospital mortality. PLR >195.8 was given an AUC value of 0.750 (95% CI 0.638-0.882, <0.001). Furthermore, an PLR >195.8 demonstrated a sensitivity of 76.5% and specificity of 78.1% for the prediction of in-hospital mortality (Table 3).

**Table 3:** Diagnostic accuracy of prognostic parameters to predict type A AAD related in-hospital mortality with the best predictive cut-offs.

	AUC	Cut-off value	Sensitivity (%)	Specificity (%)	95% CI	P-value
PLR	0.750	195.8	76.5	78.1	0.638 to 0.882	<0.001
NLR	0.746	9.74	70.6	76.8	0.623 to 0.870	<0.001

AUC=area under the curve; CI=confidence interval; NLR=neutrophil to lymphocyte ratio; PLR=platelet to lymphocyte ratio

## DISCUSSION

This study showed that NLR and PLR are useful predictors of in-hospital mortality in patients with type A AAD. Those who had high NLR and PLR on admission also had high in-hospital mortality. These findings suggest that NLR and PLR can be used as a useful clinical markers for risk classification for type A AAD.

WBC subtypes, such as NLR and PLR, are simple, widely applied, and inexpensive prognostic markers of proinflammatory state and they appear to be associated with bad clinical outcomes in various cardiovascular diseases^[6,7]^. Many studies also recommend NLR and PLR as useful predictors of outcomes in percutaneous coronary intervention, coronary artery bypass grafting, and stent restenosis^[7,9]^. In the present study, we showed that these ratios can be predictive parameters to determine the in-hospital mortality of type A AAD.

A recent study revealed the relatioship between high NLR levels and significantly high mortality rate. They also found out that the platelet count was an independent predictor of in-hospital mortality^[10]^. In this study, NLR was obtained regarding the differences between the groups to predict in-hospital mortality using ROC analysis. For the NLR, the AUC of this relationship is 0.634, the 95% CI is 0.516-0.753, and the best cut-off NLR was eight, with a sensitivity of 70% and a specificity of 53%. In our study, we found out that using a cut-off point of 9.74, the admission NLR level predicts in-hospital mortality with a sensitivity of 70.6% and a specificity of 76.8% in type A AAD with an AUC value of 0.746 (95% CI 0.623-0.870). But we found out that the platelet count had no independent prognostic factor of in-hospital mortality (*P*=0.117).

In AAD, which is a separation of the aortic wall layers, the pathogenic, genetic, environmental, and injury factors play an important role^[11]^. Previous studies have demonstrated that inflammation plays an important role in AAD^[1]^. Recent studies revealed that high PLR levels were associated with inflammation and its severity^[12,13]^. In some studies, PLR has been defined as a significant indicator of in-hospital mortality for infective endocarditis, tumors, and cardiovascular diseases^[14,16]^. Our findings revealed that at a cut-off value >195.8, PLR had an AUC of 0.750, 76.5% sensitivity, and 78.1% specificity in predicting type A AAD related in-hospital mortality.

Our study had some limitations as follows: (1) this was a single-center study that included a relatively small number of patients who were retrospectively enrolled from our database; (2) we only measured NLR and PLR on admission, so series of NLR and PLR measurements at different time points could also predict type A AAD in-hospital mortality; (3) further research is needed to understand the role of NLR and PLR in the outcome of AAD, alone or in combination with other inflammatory biomarkers.

## CONCLUSION

Admission NLR and PLR levels were important risk factors and independently associated with in-hospital mortality of type A AAD patients.

**Table:** 

Author's roles & responsibilities	
CB	Substantial contributions to the conception or design of the study; or the acquisition, analysis, or interpretation of data for the study; final approval of the version to be published
FS	Final approval of the version to be published
